# Does the heat generated by fluorescence-aided caries excavation system effect the pulp temperature of primary teeth irreversibly? An *in-vitro* evaluation of the temperature changes in the pulp chamber

**DOI:** 10.4317/jced.58785

**Published:** 2021-11-01

**Authors:** Merve Aksoy, Selin Şen, Arife Kaptan, Çiğdem Büyükkok, Firdevs Tulga-Öz

**Affiliations:** 1 Health Sciences University Faculty of Gulhane Dentistry, Department of Pedodontics, Ankara, Turkey; 2 Ankara University Faculty of Dentistry, Department of Pedodontics, Ankara, Turkey; 3 Cumhuriyet University, Faculty of Dentistry, Department of Pedodontics, Sivas, TurkeyCumhuriyet University, Faculty of Dentistry, Department of Pedodontics, Sivas, Turkey

## Abstract

**Background:**

This study aimed to analyze the effect of the Fluorescence Aided Caries Excavation (FACE) and the remaining dentin thickness on the temperature changes of the pulp chamber.

**Material and Methods:**

Freshly extracted deciduous molars and a pulpal microcirculation model were used in the study. The sample size was calculated according to power analyses (power at 90%) based on previous studies. Thus, 40 samples were needed. Standard cavities (3x3 mm) were designed to obtain a 2 mm distance through to the pulp chamber, and in each tooth (n=10), these cavities were modified to obtain 1.5 mm, 1mm, and 0.5 mm final distance through to the pulp. Coronal parts of the teeth were placed on an acrylic plate with three gaps for feeding and extraction needles and the thermocouple. The temperature changes were recorded from the initial time to 15 s and 30 s,1 min, 1.5 min, 2 min, 2.5 min, 3 min intervals.

**Results:**

The results showed that hence the thickness between cavity floor and pulp chamber was decreased, and application time of FACE was increased, an increase in temperature changes was detected. However, the recorded values were not mean to cause irreversible damages to the pulp chamber.

**Conclusions:**

The recent study showed that Face is an appropriate caries detecting system that does not affect the pulp chamber’s health, and it can be safely used in the primary teeth.

** Key words:**Caries assessment, dental caries, dental pulp, pediatric dentistry.

## Introduction

Deciduous teeth play a critical role in the growth and development of children. It is known that pulp exposures occur faster in deciduous teeth comparing to permanent dentition due to the lower hard tissue thickness of deciduous teeth. Once the pulp is affected, maintaining the teeth through permanent dentition may be difficult, and extractions may be needed (1,2). Therefore, the method’s accuracy in diagnosing and removing the infected dentin tissue in primary teeth is known as an essential issue in pediatric dentistry (3,4).

Parameters such as the hardness and color of the tissue are generally used to distinguish infected and affected dentin in conventional caries removing methods (5). However, these parameters are subjective, and determining where the preparation should finish through these observations causes a complicated situation for cavity preparation. Therefore, researchers state that a more objective technique is needed for the clinician to distinguish between infected and affected dentin (4). For this purpose, a diagnostic method called Fluorescence Aided Caries Removal (FACE) was developed in 2002. In this technique, the cavity is illuminated with blue-violet light at a wavelength of 405 nm. The operator observes the working area with 530 nm high-filter yellow lens glasses. The high pass filter blocks the purple-blue excitation light and allows a higher wavelength of fluorescent light. Therefore, infected dentin causes red fluorescence, while non-infected dentin is observed as green (6,7).

It is known that various clinical procedures, such as the pressure and heat generated by instrumentation during cavity preparation, polymerization devices, and the exothermic reaction of dental materials, may lead to an increase in the temperature of the pulp chamber. These temperature changes may cause permanent or temporary damages to the pulp chamber (8-10). The researchers held several studies to assess the effect of various parameters on the temperature changes in the pulp chamber (11-14) and the effectiveness of the FACE method on the removal of residual caries (15-19). As a result of the literature review, no study assessing the effect of the FACE application on the temperature changes of the pulp chamber was found. For this purpose, the recent study aimed to evaluate the temperature changes on the pulp chamber caused by the fluorescence light of the FACE application. The hypothesis we tested was that the application time of FACE and the remaining dentin thickness would not affect the pulp chamber’ temperature irreversibly.

## Material and Methods

-Ethical Statement 

The study protocol was conducted in line with the principles of the Helsinki Declaration, including all revisions, and with the approval of the Health Sciences University Faculty of Medicine, Gülhane, Board of Ethics (Number: 2021/103, Date: 11.03.2021). Access to data was restricted to the researchers, and informed consent was obtained from the legal representatives of all participants before tooth extraction.

-Sample Size Calculation and Experimental Groups

The sample size was calculated with a sample size calculator (Sample Size Determination in Health Studies, World Health Organization- power at 90%) based on the previous studies (11,20). Accordingly, for each group based on dentin thickness, 10 samples were required. Thus, a total of 40 samples were needed. 10 deciduous extracted sound molar teeth were used in the study (20), and four different cavity preparations were held on each of these 10 teeth. The selection of the samples was held through ICDAS (International Caries Detection and Assessment System) scores and the teeth that were sound as ICDAS scores were included in the study. Caries-free deciduous teeth extracted due to physiological root resorption were collected and cleaned using a periodontal scaler. The samples have remained in 0.1% thymol until the experiment date, and the maximum time was detected as one month.

-Cavity Preparation

The enamel of the occlusal part of the extracted teeth was abraded, and roots were removed under water cooling using a carbon disk bur from 2 mm apical of the cementoenamel junction. The pulp residues in the pulp chambers were removed with hand tools, and the pulp chamber was irrigated with 5.25% sodium hypochlorite and distilled water. Cavities were prepared by using a diamond fissure bur (no:015, Romi Diamond, Israel) with a dentin thickness of 2 mm, 1.5 mm, 1 mm, and 0.5 mm between the cavity base and the pulp ceiling, and the accuracy of this thickness was confirmed by an orthodontic caliper (Iwanson, Pearson). The cavity was prepared and measured with the remaining dentin thickness of 2 mm, and then the cavity was deepened till the remaining dentin thickness was 0.5 mm, in the same sample. In that way, four assessments could be done in one tooth sample, so the standardization was obtained. The width of the cavity borders was standardized by affixing 3x3 mm labels to the occlusal surface, and the outside surface of the label was painted with nail polish. The cavity preparation was finished by the polished area.

-Pulpal microcirculation model

**Figure 1:**
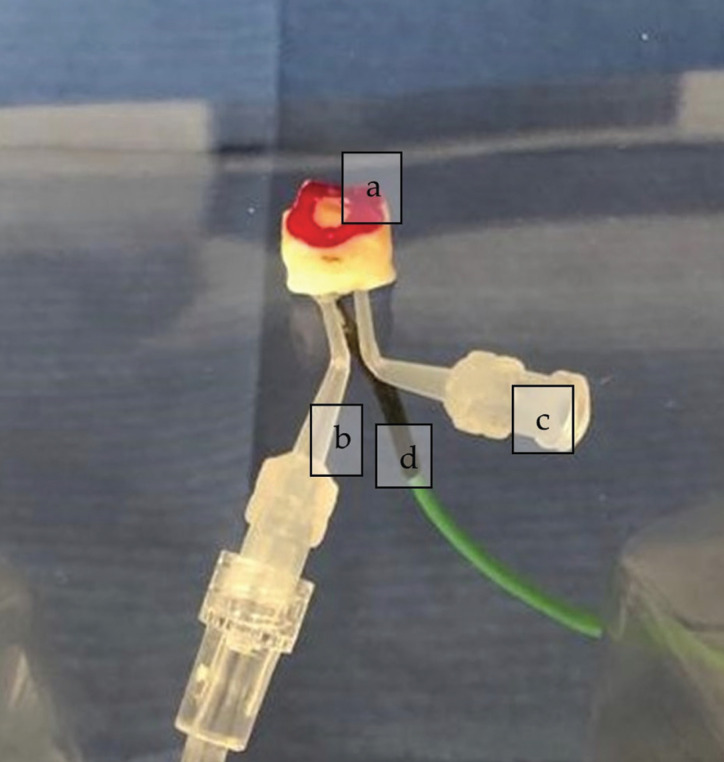
a) Detection of the cavity borders with nail polish. b) Needle for feeding by water. c) Needle for extraction of the excess water. d) The probe of the J type thermocouple.

A pulpal micro-circulation model designed by Savas *et al*. (21) was used in the recent study. Pulpal microcirculation was achieved by using deionized water with an infusion set. Teeth were placed and fixed by a light-curing adhesive cement (Panavia, Kuraray) on an acrylic plate, and three gaps were performed on it. Two of them were for the feeding and the extraction needles of deionized water at room temperature. Into the third gap, the thermocouple was placed to assess the pulp chamber’s temperature changes. The J-type thermocouple (Schneider) was placed into a thermal paste (Rampage TM-130) which was applied on the upper surface of the pulp chamber (Fig. 1). In this design, while deionized water was led into the pulp from one way, the excess water was drained by another similar to *in-vivo* conditions. The flow rate of saline solution was determined as 1 ml/sec, and intra-pulpal pressure was adjusted to 15 cm H2O according to previous studies (11,22).

**Figure 2:**
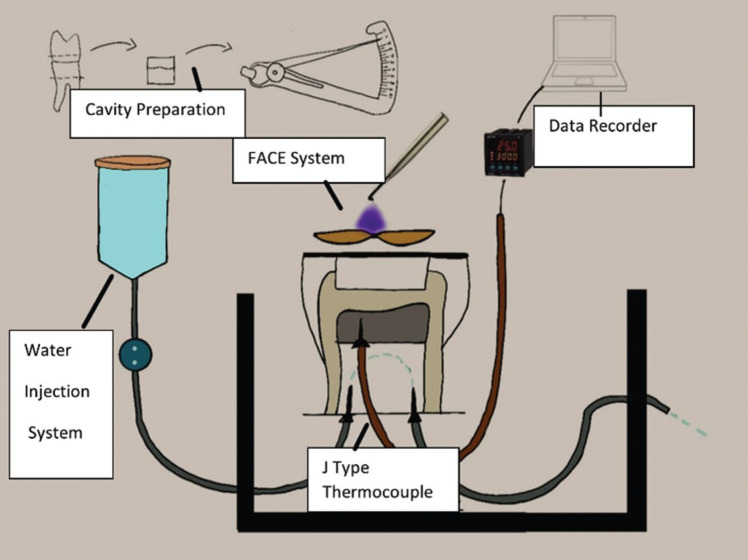
Study Design. Teeth were cut from 2 mm apical of the cemento-enamel junction. Three gap formation was performed on the acrylic plate for needles of extraction- feeding probes and the thermocouple. The temperature changes were detected by the data recorder connected to the thermocouple.

Fluorescence light was applied to each sample, and the initial temperature was recorded using a data logger (PID EMKON Quadro, Turkey) connected to the thermocouple. For each sample, the temperature values before fluorescence light application were calculated at each distance. Then, the values at 30 seconds, 1 min, 1.5 min, 2 min, 2.5 min, and 3 min were measured. These measurements were repeated 3 times to get reliable results, and the mean values were noted. The differences (ΔT) between the initial mean temperature values (T0) and the mean values detected at each distance (Tx) were calculated (ΔT= Tx - T0). The study design and pulpal microcirculation model was schematized in Figure 2.

-Statistical analyses

The data obtained in this study were analyzed with IBM SPSS 21 package program.

Shapiro Wilk’s and Kolmogorov Smirnov Tests were used for the analyses. While examining the differences between groups, the Kruskal Wallis-H Test was used if the variables were not from the normal distribution. If significant differences were observed in the Kruskal Wallis-H Test, the groups with differences were determined by Post-Hoc Multiple Comparison Test. 0.05 was used as the significance level, and it was stated that there is a significant difference when *p* <0.05, and there is no significant difference if *p*> 0.05.

## Results

The data obtained by the statistical analyses of the recent study are shown in Table 1, Table 2 and Table 3.

**Table 1:**
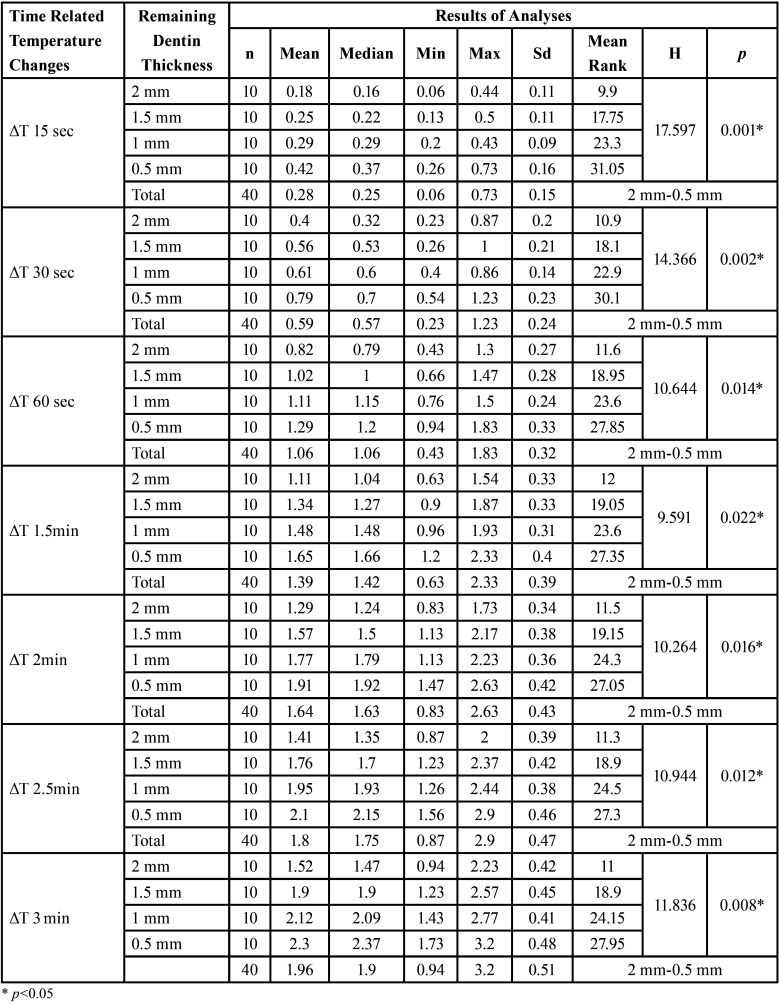
Results of analyses of the temperature changes at any distance during the measurement.

**Table 2:**
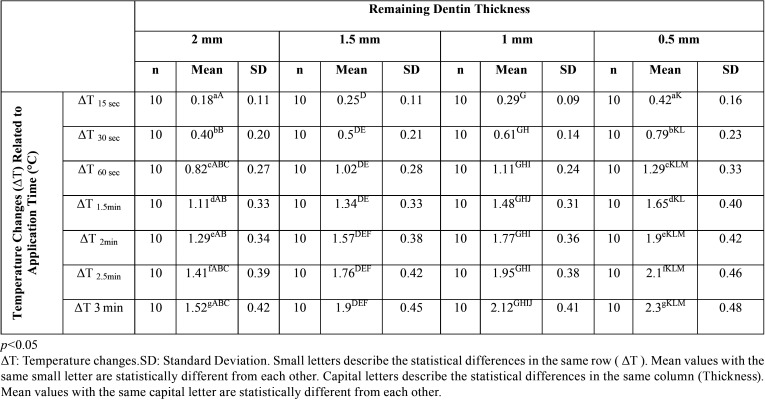
Statistical analyses of pulpal temperature changes in each dentinal thickness and at different measurement time of FACE application (Kruskal Wallis H Test).

**Table 3:**
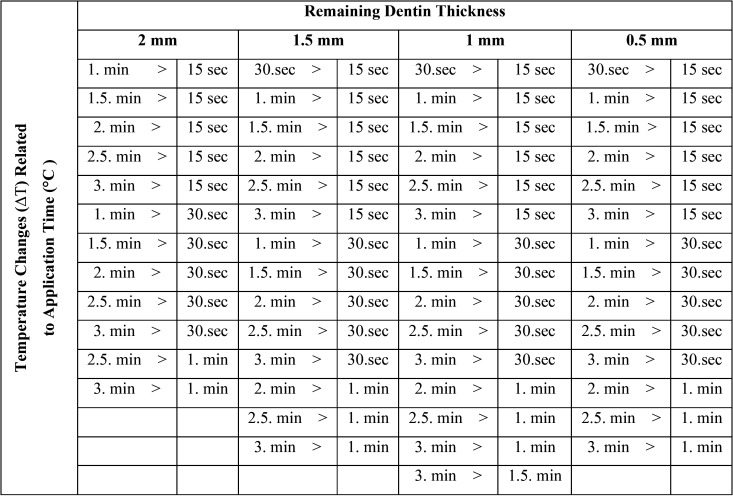
Statistically different temperature changes detected at different measurement times and in each dentinal thickness.

In Table 1, temperature changes related to different dentinal thicknesses were shown. According to these results, the temperature changes measured at 0.5 mm dentin thicknesses were statistically lower than the values detected at 2 mm dentin thicknesses for each measurement at all time intervals (*p*<0.05).

**Figure 3:**
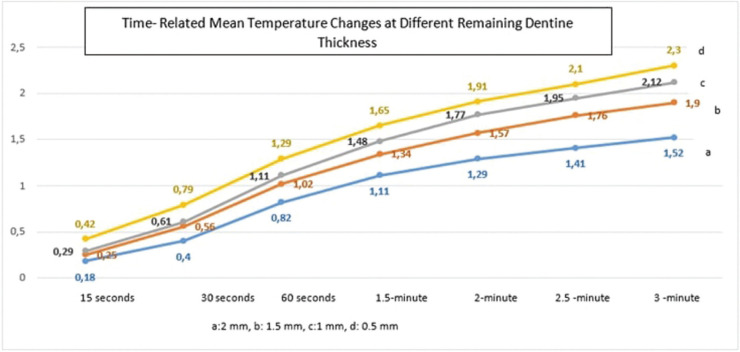
Time-related temperature changes at different remaining dentin thicknesses. As the cavity was deepened through to the pulp chamber and the application time of FACE was increased, the mean temperature changes were increased as well. The maximum temperature change was detected at 2.3°C in 0.5 mm dentin thickness at 3 minutes of application.

The relation between the pulp temperature changes and the remaining dentin thicknesses is shown in Table 2. Accordingly, in all dentinal thicknesses (2 mm, 1.5 mm, 1 mm, 0.5 mm), the pulp temperature changes measured each time differed statistically significantly (*p*<0.05). The pulp temperature changes of the first 15 sec were different from the values detected at 30 sec, 60 sec, 1.5 min, 2 min, 2.5 min, and 3 min measurements in all dentinal thicknesses except the 2 mm group (*p*<0.05). Temperature changes at 30 seconds were statistically different from the other intervals in all dentinal thicknesses (*p*<0.05). Furthermore, at 1 min intervals, temperature changes were detected differently from 2.5 and 3 min measurements in all dentinal thicknesses (*p*<0.05). In the group of 1mm thickness, the temperature changes detected at 3 min were higher than the values assessed at 1.5 min measurements (*p*<0.05), ( Table 2, Table 3), (Fig. 3).

## Discussion

The end line of cavity preparation remains to be the main problem for clinicians. Due to dentinal tissue’s high permeability in deciduous teeth, bacterial remains may lead to seconder caries and pulpal infections that end in pulp necrosis (23). Recently, FACE has been developed for this area to determine where to finish cavity preparation. In this system, it is possible to perform a cavity preparation, causing less hard tissue loss due to the minimal invasive potential of the method. Although the effect and reliability of this method have been proved in previous studies (16,24,25), it is remained to be unknown if this method leads to irreversible pulpal damages due to the heat generated by the FACE applications.

Infrared cameras, calorimeters, different thermal analyses, and thermocouples have been used to examine the heat changes in dental tissue. The infrared method aims to examine the changes on the surface directly affected by the heat. It is possible to indirectly analyze the heat conducted through the pulp chamber using a thermocouple (20). Therefore, in the recent study, J type thermocouple was used to assess the intra-pulpal temperature changes. Based on the knowledge that pulpal microcirculation plays an essential role in eliminating the pulp’s temperature increase, the mechanism resembling pulpal microcirculation was used (21). In that way, more reliable results were aimed to obtain. The drop rate of deionized water in the injection system was set at 1 ml/ min (11,22). In some of the studies, the saline solution and deionized water were placed in a thermal bath at 37°C (11,22,26), and the room temperature was chosen in the others (20,21). In the recent study, deionized water at room temperature was chosen to be used.

Cavity walls were standardized by affixing 3x3 mm stickers on the tooth’s surface (11), and the deepness of the cavity through the pulp chamber was obtained on the same tooth for each measurement. Permeability of dentin may differ from one tooth to another due to the dentin thickness, mineralization of hard tissue, the physiological root resorption of the tooth, and the width of dentinal tubules (11). To eliminate these factors related to each tooth, we have prepared the cavities on the same samples for each thickness. Thus, the measurement standardization has been achieved by gradually reducing the dentin thickness from 2 mm to 1.5 mm, 1 mm, and 0.5 mm on the same tooth and keeping the initial temperature values at approximate values.

Bacterial metabolites that cause red fluorescence produce less fluorescence in the long-term illumination of the teeth with the FACE method. This long application can result in inadequate removal of infected dentin tissue. According to this, it is recommended that the illumination time of the cavity with FACE should not be more than 3 minutes (27). For this reason, the light application time in the recent study was planned to be 3 minutes at most.

The recent study showed that as the cavity was deepened and the application time of FACE was increased, the temperature changes detected in the pulp chamber were found to rise statistically significantly (*p*<0.05). The pulpal temperature changes were significantly increased from the initial time to 1.5 min application in all dentin thicknesses. They remained to increase through the time at 2 min, 2.5 min, 3 min applications. The recent study results were the same as the previous studies in which pulpal temperature changes were detected as the cavity preparation was deepened through to the pulp (11,28).

Although no study has examined the pulpal temperature changes related to FACE applications on deciduous teeth, the effect of exothermic polymerization reactions and the curing techniques were investigated in a few studies. Zach and Cohen (8) detected that the critical degree that may lead to pulpal damages was 5.6 °C. The highest temperature change was detected as 2.30 °C at 0.5 mm dentin thickness, so the temperature changes of the pulp chamber during the application of FACE were detected under this critical degree in the recent study. According to this result, the hypothesis we tested was accepted.

In a previous study held by Kaptan and Büyükkok (11), temperature increases in the pulp chamber of primary teeth during polymerization of different glass ionomer-based restorative materials were evaluated in thicknesses 1 mm and 2 mm remaining dentin. In the result of this study, the heat generated and conducted through the pulp chamber by the polymerization of the glass ionomer materials was detected higher in 1mm dentin thickness statistically significantly than the values detected in 2 mm remaining dentin thickness in accordance with the recent study we held.

An *ex-vivo* study aimed to determine the pulp response to the heat generated by light-curing units. In this study, different thickness of tooth slices was exposed to light-curing units, and temperature changes were recorded at increasing time intervals at 10 s, 20 s, 30 s, 40 s. As a result of the study, an increase was observed in the heat generated and conducted through to the pulp, accordingly to the decrease in the dentin thickness and the increase in the time of light application, and these results were found to be consistent with the data obtained in the recent study (28).

## Conclusions

In conclusion, according to the recent study results, caries detection by FACE in primary teeth, in different remaining dentinal thicknesses, could be a safe method following the pulpal temporal changes. It is possible to say that when FACE is used in primary teeth as a caries detection system, the heat generated by the method does not affect the pulp temperature irreversibly, considering the recent study results. Although the number of studies is insufficient in this area, it is possible to say that FACE can be safely used as a caries detection system in the primary tooth. However, further studies investigating the relation between the pulpal temperature changes and FACE application should be performed in the future.
